# Clinical outcomes of monitored anesthesia care vs. general anesthesia in transfemoral transcatheter aortic valve implantation: a single-center retrospective study

**DOI:** 10.3389/fcvm.2025.1652045

**Published:** 2025-11-25

**Authors:** Haoran Zhang, Tao Chen, Bo Yu, Donghui Zhang

**Affiliations:** 1 Department of Cardiology, The Second Affiliated Hospital of Harbin Medical University, Harbin, China; 2 The Key Laboratory of Myocardial Ischemia, Chinese Ministry of Education, Harbin, China

**Keywords:** transcatheter aortic valve implantation, monitored anesthesia care, minimalist approach, transfemoral, aortic stenosis

## Abstract

**Background:**

Transcatheter aortic valve implantation (TAVI) has become a preferred treatment for severe aortic stenosis in high-risk patients. In China, general anesthesia (GA) remains the standard; however, monitored anesthesia care (MAC) offers a less-invasive alternative. In this study, we compared the outcomes between MAC and GA in transfemoral TAVI.

**Methods:**

We retrospectively analyzed the data from 106 consecutive patients (54 MAC, 52 GA) who underwent TAVI at a The Second Affiliated Hospital of Harbin Medical University from January 2021 to November 2023. MAC involved administration of a combination of local anesthesia with dexmedetomidine/remifentanil sedation, whereas GA involved endotracheal intubation. We compared procedural metrics, complications, and lengths of hospital stays.

**Results:**

The baseline characteristics were comparable between the groups (mean age: 70.3 ± 7.3 years, 46% with reduced ejection fraction). The MAC group showed shorter procedure times (102 ± 25 vs. 145 ± 42 min, *p* < 0.0001) and hospital stays (10.5 ± 3.7 vs. 14.1 ± 5.1 days, *p* < 0.0001), compared with the GA group. Safety outcomes were similar between the groups: 30-day mortality (5.8% vs. 7.4% in the MAC and GA groups, respectively, *p* = 0.734), stroke (1.9% in both groups), and major vascular complications (3.8% vs. 5.6%). The GA group had higher valve-in-valve rates (13% vs. 1.9%, *p* = 0.024) and postoperative hypotension (4 vs. 0 patients), compared with the MAC group. Pacemaker implantation was more frequent in the MAC group vs. the GA group (13% vs. 3.8%, *p* = 0.229).

**Conclusions:**

The use of MAC for TAVI is comparably safe to using GA, with potential advantages in recovery speed and resource utilization. A heart team approach, including cardiac anesthesiologists, is critical for optimal patient selection.

## 1 Introduction

The treatment of aortic stenosis in older patients with high surgical risk in China has improved with the introduction of transcatheter aortic valve implantation (TAVI). TAVI provides a safer and more effective treatment option for patients (1, 2). It has become the preferred treatment for patients at high or prohibitive surgical risk. The number of procedures conducted has been increasing annually (3). TAVI can be conducted under both general anesthesia (GA) and local anesthesia/monitored anesthesia care (MAC). MAC is defined as cardiovascular and respiratory monitoring by a qualified anesthesiologist who may or may not administer sedation. For older patients, GA may increase the risks of cardiac, cerebral (4), and pulmonary complications (5). In such cases, using local anesthesia/MAC combined with mild analgesic drugs presents an attractive alternative to GA, helping to avoid these complications. With growing clinical experience and advancements in transcatheter techniques, some operators are promoting an ultra-minimalist TAVI approach (6). This method involves performing transfemoral TAVI under local anesthesia with mild analgesics and fluoroscopic guidance.

Currently, approximately 95% of TAVI procedures in China are performed under GA (7). GA is typically administered by anesthesiologists who are experienced in treating patients undergoing conventional cardiac surgery. There are significant regional differences in anesthesia approaches between China and Europe/America. The proportion of MAC and GA usage in some European cardiac centers is nearly equal, with the use of MAC showing an increasing trend annually (8, 9). In our center, we conducted a retrospective study comparing the outcomes between MAC and GA in TAVI procedures performed between 2021 and 2023.

## 2 Materials and methods

Clinical data were retrospectively obtained from consecutive adult patients who underwent transfemoral TAVI between January 2021 and November 2023 at the Heart Center of the Second Affiliated Hospital of Harbin Medical University. In this study population, we included patients with severe aortic stenosis requiring aortic valve replacement, defined as an aortic valve area <1 cm^2^, a peak aortic velocity >4 m/s, or a mean pressure gradient >40 mmHg (10).

### 2.1 Anesthesia management

The same qualified cardiothoracic anesthesiologist administered all anesthesia procedures. Standard monitoring was applied to all patients, including electrocardiography and pulse oximetry. Before initiating general anesthesia in the GA group or sedation in the MAC group, an arterial catheter (right radial artery) and a central venous catheter (median cubital vein) were placed.

In the GA group, anesthesia was induced using etomidate (0.15–0.3 mg/kg), sufentanil (0.25–0.5 μg/kg), and lidocaine (1.5 mg/kg). All patients were then intubated with an endotracheal tube. Anesthesia was maintained with continuous infusions of propofol, sufentanil, and cisatracurium.

In the MAC group, most patients received oxygen through a nasal cannula, with noninvasive ventilator mask ventilation utilized when necessary. Sedation was achieved using continuous infusions of dexmedetomidine [0.1–0.5 μg/(kg·h)] and remifentanil [2–4 μg/(kg·h)], supplemented with etomidate (0.15–0.3 mg/kg) as needed. Low-dose propofol [2–6 mg/(kg·h)] was administered during skin incision, pacing, balloon dilation, and valve deployment. Oxygen mask support was provided as required to maintain airway patency.

The mean arterial pressure was maintained above 90 mmHg (1 mmHg = 133.3 Pa) throughout the procedure. Intravenous catecholamines (dopamine) were administered to treat hypotension, whereas sodium nitroprusside or urapidil was used to manage hypertension. All patients were transferred to the cardiac care unit after the procedure. No patients in the MAC group required conversion to general anesthesia during the procedure.

### 2.2 TAVI management

The same team of cardiologists conducted all the TAVI procedures. The VenusA-Valve (Venus MedTech, Hangzhou) or TaurusElite (Peijia Medical, China) devices were implanted in all patients. The cardiologists determined the selection of valve size based on the transthoracic echocardiography (TTE) and dual-source computed tomography findings.

Two ProGlide percutaneous vascular closure devices (Abbott Vascular, USA) were pre-embedded to close the femoral artery access site before deploying the valve. In most cases, balloon valvuloplasty was performed under rapid ventricular pacing to predilate the native valve. The new valve was then implanted under fluoroscopic guidance. Aortic root angiography and TTE were used to confirm proper valve positioning and functionality.

### 2.3 Statistical analyses

Statistical analyses were performed using the SPSS software package (version 26.0). Continuous variables were expressed as mean ± standard deviation (SD) and compared using independent samples t-tests. Categorical variables were analyzed using Fisher's exact test or the *χ*^2^ test, as appropriate. A *p*-value <0.05 was considered statistically significant. All values are shown as mean ± SD unless stated otherwise.

## 3 Results

In this study, among 106 patients who underwent TAVI procedures in the cardiac catheterization laboratory of Harbin Medical University between January 2021 and November 2023, 2 were excluded owing to incomplete data. The remaining 106 patients comprised 52 (49.1%) who received GA and 54 (50.9%) who underwent the procedure under sedation with MAC.

**Figure 1:**
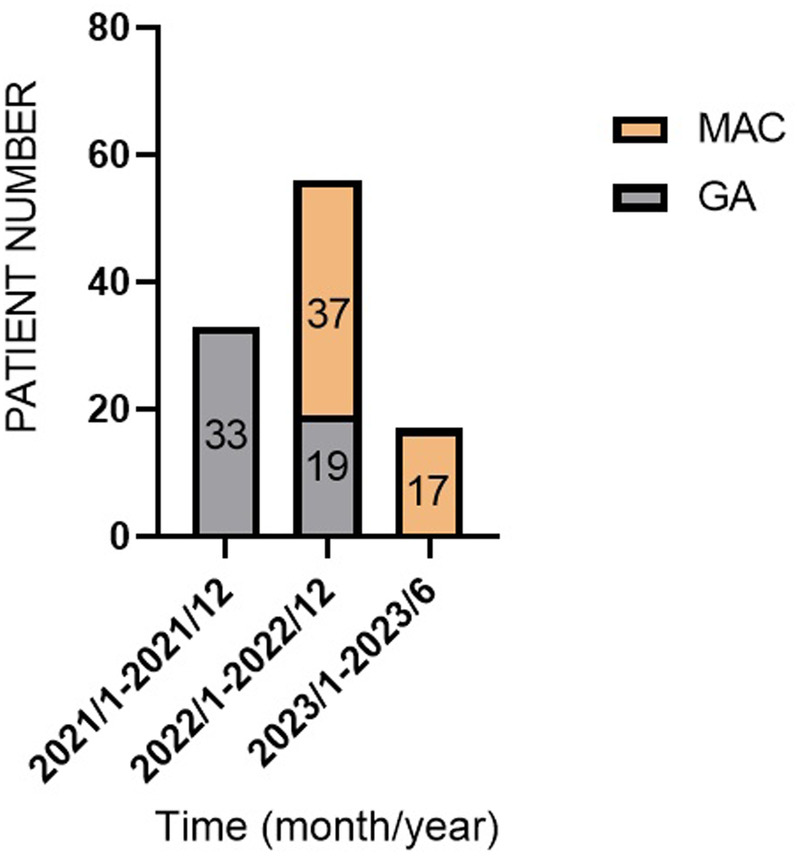
Flowchart of patient enrollment and group allocation (2021–2023).

A temporal shift in anesthesia preference was observed (Figure 1). GA was predominantly used between January 2021 and December 2021. However, most of the patients received MAC between January 2023 and June 2023. Notably, there were no cases of failed sedation, and no patients required conversion to GA due to procedural complications.

### 3.1 Baseline characteristics

Overall, 106 patients met the inclusion criteria and underwent transfemoral TAVI. The procedures were performed under MAC in 54 patients (50.9%) and under GA in 52 patients (49.1%). The mean age was 71.2 ± 7.3 years in the MAC group and 69.5 ± 7.3 years in the GA group. Table 1 shows the baseline characteristics, with the two groups showing comparable demographic profiles.

**Table 1:** Baseline patient characteristics.

Characteristics	GA (*n* = 52)	MAC (*n* = 54)	Overall (*n* = 106)	*p*-value
Age	69.5 ± 7.3	71.2 ± 7.3	70.4 ± 7.3	0.231
Male	32 (61.6%)	29 (53.7%)	61 (57.5%)	0.415
NYHA III	14 (24.1%)	13 (26.9%)	27 (25.5%)	0.736
NYHA IV	16 (24.1%)	13 (30.8%)	29 (27.4%)	0.440
Hypertension	18 (34.6%)	23 (42.6%)	41 (39.6%)	0.335
Dyslipidemia	7 (13.5%)	8 (14.8%)	15 (15.1%)	0.819
IHD	20 (38.5%)	15 (27.8%)	35 (34.0%)	0.172
CHF	6 (48.1%)	3 (57.7%)	9 (52.8%)	0.178
Stent implantation	7 (13.5%)	9 (16.7%)	16 (15.1%)	0.601
CVA	8 (15.4%)	9 (16.7%)	17 (16.0%)	0.837
TIA	1 (1.9%)	6 (11.1%)	7 (6.6%)	0.046
PVD	3 (5.8%)	9 (16.7%)	11 (11.3%)	0.056
Smoking	14 (26.9%)	8 (14.8%)	22 (19.8%)	0.066
COPD	2 (3.8%)	4 (7.4%)	6 (5.7%)	0.383
Diabetes	16 (30.8%)	13 (24.1%)	29 (28.3%)	0.367
CRF	0 (0.0%)	4 (7.4%)	4 (3.8%)	0.044
Arrhythmias	14 (26.9%)	7 (13.0%)	21 (20.8%)	0.030
Thyroid abnormalities	2 (3.8%)	1 (1.9%)	3 (2.8%)	0.449
Rheumatism	1 (1.9%)	1 (1.9%)	2 (1.9%)	0.975
Renal dysfunction	6 (11.5%)	2 (3.7%)	8 (8.5%)	0.055
Liver dysfunction	1 (1.9%)	2 (3.7%)	3 (2.8%)	0.544
Antiplatelet drugs	9 (17.3%)	12 (22.2%)	21 (19.8%)	0.06
Antihypertensive drugs	14 (26.9%)	25 (46.2%)	39 (36.7%)	0.002
Antiarhythmics drugs	0 (0.0%)	2 (3.7%)	2 (1.9%)	0.160
Antidiabetic drugs	12 (23.0%)	12 (22.2%)	24 (22.6%)	0.049
Respiratory drugs	0 (0.0%)	6 (11.1)	6 (5.6%)	0.049

IHD, ischemic heart disease; CHF, congestive heart failure; CVA, cerebrovascular accidents; TIA, transient ischemic attack; PVD, peripheral vascular diseases; COPD, chronic obstructive pulmonary disease; CRF, chronic renal failure.

Approximately half of the patients showed symptoms of heart failure and were classified as New York Heart Association class III/IV (Table 2). The most prevalent comorbidity was hypertension (39.6%), followed by ischemic heart disease (33.96%), diabetes mellitus (28.3%), and arrhythmias (20.8%). We observed reduced ejection fraction in 36.5% of the patients in the GA group compared with 46.3% of those in the MAC group.

**Table 2:** Preoperative echocardiographic parameters.

Variable/complication	GA (*n* = 52)	MAC (*n* = 54)	Overall (*n* = 106)	*p*-value
Left ventricular size	48.78 ± 1.04	48.78 ± 0.95	49.69 ± 0.71	0.626
Interval size	12.63 ± 0.24	13.27 ± 0.32	12.94 ± 0.20	0.751
Pressure gradient	65.75 ± 3.55	82.43 ± 4.93	73.93 ± 3.11	0.153
Left ventricular ejection fraction	55.05 ± 1.73	52.92 ± 1.50	54.42 ± 1.24	0.849
>55%	33 (31.1%)	29 (27.4%)	72 (67.9%)	0.069
45%–55%	12 (11.3%)	8 (7.5%)	20 (19.0%)	
30%–44%	5 (4.7%)	14 (13.2%)	19 (18.0%)	
<30%	4 (3.8%)	1 (0.9%)	5 (4.8%)	
Aortic velocity	4.65 ± 0.10	4.67 ± 0.13	4.66 ± 0.08	0.132

### 3.2 Perioperative variables

Table 3 shows the perioperative variables and data on complications. The MAC group showed a significantly shorter total procedure time than the GA group (102.35 ± 25.35 min vs. 145.46 ± 41.65 min, *p* < 0.0001). The 95% confidence interval for the difference in procedure time was [29.74, 56.46] min, and the effect size (Cohen's *d*) was 1.25, indicating a large effect. Similarly, the MAC group had shorter average hospital stays than the GA group (10.46 ± 3.74 days vs. 14.06 ± 5.08 days, *p* < 0.0001). The 95% confidence interval for the difference in hospital stay was [1.88, 5.32] days, with a moderate effect size (Cohen's *d* = 0.81).

**Table 3:** Periprocedural variables and complications.

Variable/complication	GA (*n* = 52)	MAC (*n* = 54)	Overall (*n* = 106)	*p*-value
Operation time (min)	145.5 ± 41.7	102.4 ± 25.4	123.5 ± 40.4	<0.0001
Length of hospital stay (d)	14.1 ± 5.1	10.5 ± 3.7	12 ± 4.8	<0.0001
Postoperative intubation	1 (1.9%)	0 (0.0%)	1 (0.9%)	0.148
Emergency surgery during perioperative period	1 (1.9%)	3 (5.6%)	4 (3.8%)	0.293
Thirty-day mortality	3 (5.8%)	4 (7.4%)	7 (6.6%)	0.734
Hypotension	4 (7.7%)	0 (0.0%)	4 (3.8%)	0.004
Pacemaker implantation	2 (3.8%)	7 (13.0%)	9 (8.5%)	0.092
ECMO	1 (1.9%)	4 (7.4%)	5 (4.7%)	0.183
Pulmonary edema	1 (1.9%)	0 (0.0%)	1 (0.9%)	0.148
Pneumonia	2 (3.8%)	5 (9.3%)	7 (6.6%)	0.223
Respiratory failure	0 (0.0%)	0 (0.0%)	0 (0%)	
Hemorrhage	2 (3.8%)	4 (7.4%)	6 (5.7%)	0.383
Control of blood products	0 (0.0%)	2 (3.7%)	2 (1.9%)	0.16
Fever	3 (5.8%)	4 (7.4%)	7 (6.6%)	0.701
Viv	7 (13.0%)	1 (1.9%)	8 (7.6%)	0.024
Acute renal injury	0 (0.0%)	1 (1.9%)	1 (0.9%)	0.323
Nervous system	1 (1.9%)	2 (3.7%)	3 (2.8%)	0.544
Local vascular injury	1 (1.9%)	3 (5.6%)	4 (3.8%)	0.293

ECMO, extracorporeal membrane oxygenation; Viv, valve-in-valve.

Regarding perioperative outcomes, MAC group showed 30-day mortality rates that were comparable to those of the GA group (5.8% vs. 7.4%, *p* = 0.734, Table 3). Perioperative echocardiographic data are shown in Table 2 and demonstrated no significant intergroup differences in left ventricular function or valve gradients. TAVI performed under MAC did not increase the risk of adverse events compared with that performed under GA, with no significant differences observed in stroke rates or major vascular complications. The permanent pacemaker implantation rate was 13.0% in the MAC group vs. 3.8% in the GA group (*p* = 0.229). Table 3 shows the distribution of prosthesis types and pacemaker implantation rates following anesthesia technique.

Notably, the GA group showed a significantly higher incidence of valve-in-valve implantation than did the MAC group (13.0% vs. 1.9%, *p* = 0.024, Table 3). Notably, four patients in the GA group experienced postoperative hypotension, whereas no such events were observed in the MAC group. Regarding complications, the GA group showed a reintubation rate of 1.90% postoperatively, whereas no patients in the MAC group required reintubation. The incidence of pneumonia was 3.8% in the GA group vs. 9.3% in the MAC group (*p* = 0.223, Table 3). However, none of these differences were significant (*p* = 0.148 for reintubation rate comparison).

## 4 Discussion

With accumulating surgical experience and advancements in transcatheter devices, transfemoral TAVI has become feasible under both GA and MAC. This development has led some operators to propose the concept of minimalist TAVI (6). In this study, all 106 enrolled patients successfully underwent valve implantation. These older patients (mean age: 70 years) significantly benefited from the TAVI procedure, especially through the transfemoral approach, which offers minimal invasiveness and rapid recovery (7).

At our center, the use of MAC refers to conducting TAVI under local anesthesia combined with mild analgesic agents without the need for transesophageal echocardiography guidance or endotracheal intubation. Valve positioning and deployment are conducted solely under fluoroscopic guidance. Based on our experience, we use dexmedetomidine as the primary sedative during MAC. Supplemental low-dose propofol is administered only during critical procedural stages, including skin incision, ventricular pacing, balloon valvuloplasty, and valve deployment to deepen sedation when necessary. This approach allows most patients to remain asleep throughout the procedure while effectively managing pain responses and alleviating discomfort caused by rapid pacing-induced hypotension.

Theoretically, MAC is suitable for patients with relatively preserved cardiac function who can maintain a supine position without movement and have no airway difficulties. However, GA may be more appropriate for patients requiring prolonged procedures or those unable to remain still, as it involves mechanical ventilation and anesthetic agents that can modulate hemodynamics while enabling transesophageal echocardiography for enhanced intraoperative imaging.

In this study, we demonstrated that MAC has good tolerability even in patients with reduced ejection fraction, compared with GA. Notably, four patients in the MAC group successfully underwent TAVI with extracorporeal membrane oxygenation support. These patients had severe cardiac dysfunction, where the use of GA might have posed challenges, including difficult induction, prolonged procedure time, and extubation complications. Furthermore, mechanical ventilation was directly associated with increased pneumonia risk, especially in older patients (8).

Compared with GA, MAC shows several distinct advantages. Most importantly, it preserves the patient's ability to provide immediate feedback during the procedure. This advantage was exemplified in our study when a patient experienced sudden chest pain after valve deployment. The symptom prompted urgent angiography, which revealed a coronary obstruction, enabling timely intervention. Furthermore, because patients remained conscious under MAC, continuous anesthesiologist monitoring was still required, ensuring patient safety, while significantly reducing staff workload and shortening both procedural duration and hospital stay. Collectively, these factors contribute to lower medical costs and decreased risks of hospital-acquired infections and other complications associated with prolonged hospitalization. The combination of enhanced safety monitoring and improved operational efficiency makes MAC an increasingly preferred approach for suitable TAVI candidates (9). The observed difference in total procedure time primarily originates from the additional time required for anesthesia induction and extubation when using GA. The core surgical duration is fundamentally determined by the operator's technical proficiency and the patient's individual anatomical and pathological characteristics; nevertheless, the extended overall procedure time associated with GA inevitably leads to increased utilization of medical resources and higher procedural costs. This creates significant implications for healthcare resource allocation.

Regarding perioperative complications, the GA group in this study showed a significantly higher incidence of postoperative hypotension (*p* < 0.05), which may be attributed to the vasodilatory effects of general anesthetic agents. The initially elevated valve-in-valve implantation rate was potentially associated with the early adoption of first-generation retrievable valves in our center, as their material composition and skirt design characteristics might have contributed to an increased incidence of paravalvular leakage (11). Moreover, factors such as valve size and implantation depth could also have influenced the valve-in-valve implantation rate, as oversized valves may exert excessive pressure on the aortic annulus, leading to conduction disturbances, while undersized valves may result in paravalvular leakage. Furthermore, deeper implantation depths could compress the cardiac conduction system, increasing the risk of conduction block or the need for permanent pacemaker implantation (12, 13). Notably, after transitioning to second-generation valves, a marked reduction in valve-in-valve requirements was observed. This reduction may be attributed to the optimization of valve size and advancements in surgical techniques, which have improved the precision of valve implantation and reduced the need for valve-in-valve procedures.

Several studies show that TEE monitoring may contribute to the observed differences in paravalvular leakage and pacemaker implantation rates between the groups (14). Both two-dimensional TTE and TEE consistently underestimate aortic valve annular dimensions, compared with three-dimensional TEE. In this study, TEE was not used in the MAC group. the MAC group showed a higher numerical trend of permanent pacemaker implantation, compared with the GA group; however, this difference was not significant (*p* > 0.05).

The transition from GA to MAC represents a significant advancement toward minimally invasive TAVI. The “minimalist approach” to TAVI implementation can obviate the routine presence of anesthesiologists and reduce associated labor costs (7). However, regardless of the anesthesia technique used, experienced cardiac anesthesiologists must supervise perioperative management to ensure patient safety and procedural success. Our study showed that this approach reduced procedural duration and shortened hospital stays, leading to significant cost savings that may substantially impact overall healthcare expenditures.

However, as a retrospective study, the patient selection for MAC vs. GA may have been influenced by unmeasured factors such as operator preference, patient comorbidity, and anatomical complexity. These factors could introduce confounding bias, which may affect the interpretation of the results. We acknowledge these limitations and recognize that they may have impacted the validity of our findings. Future prospective studies or studies utilizing multivariate analysis to adjust for these confounders would help to further elucidate the benefits and risks of MAC compared to GA.

In conclusion, transfemoral TAVI conducted under MAC shows comparable safety and efficacy to GA, with similar clinical outcomes and a trend toward faster recovery. A dedicated multidisciplinary “valve heart team” approach, including cardiac anesthesiologists, remains essential for comprehensive perioperative patient management.

## Data Availability

The raw data supporting the conclusions of this article will be made available by the authors, without undue reservation.
